# A Study on the Properties of Blended Recycled Micro Powder Concrete and Insulation Boards Produced via Microbial Foaming

**DOI:** 10.3390/ma19102149

**Published:** 2026-05-20

**Authors:** Hai-Yan Zhang, Gui-Qiang Li, Hu-Bin Bai, Yu-Jiao Zhang, Hui Rong, Xiang-Guo Li

**Affiliations:** 1 China Testing & Certification International Group Xi’an Co., Ltd., Xi’an 710061, China; zhy0921a@163.com (H.-Y.Z.); baiskoay@163.com (H.-B.B.);; 2 School of Materials Science and Engineering, Wuhan University of Technology, Wuhan 430070, China; 3 College of Soil and Water Conservation Science and Engineering, Northwest A&F University, Xianyang 712100, China; 4 School of Materials Science and Engineering, Tianjin Chengjian University, Tianjin 300384, China; 5 Joint Research Center for Chinese and Polish Building Green Functional Materials and Technology, Tianjin Chengjian University, Tianjin 300384, China; 6 Tianjin Key Laboratory of Functional Materials for Building Green Materials, Tianjin 300384, China

**Keywords:** mixed regenerated micro powder, microbial foaming agent, foam insulation board, thermal conductivity

## Abstract

Promoting construction waste utilization, this study explores using 30% mass fraction recycled micro powder (brick/concrete/hybrid) to replace cement in A03 foam concrete, as well as microbial foaming agents for insulation boards. The results show that hybrid micro powder foam concrete achieved higher compressive strength (0.7 MPa, 0.65 MPa) than pure brick (0.54 MPa) and concrete powder (0.61 MPa). For 30% hybrid micro powder insulation boards (brick:concrete ratios 2:8–8:2), when the ratio is 4:6, their performance meets JC/T 2200-2013 standards. At this point, the compressive strength is 0.43 MPa, the drying shrinkage is 0.29 mm, and the thermal conductivity 0.062 is W/(m·K). As the proportion of recycled brick powder increases, the material properties first improve and then decline, indicating that the proportion of recycled brick powder has a significant impact on the material’s overall performance; within an appropriate range, optimal performance can be achieved.

## 1. Introduction

Foam concrete (FC) is a lightweight concrete with a density of 300~1800 kg/m^3^ [1], which is formed by mixing a foam agent and cementitious material and has a random pore structure. As an inorganic fireproof material, foam concrete has been widely used in the field of building insulation due to its lightweight and economical characteristics. Compared with organic thermal insulation materials, which are flammable, are prone to falling off, have a short service life, and often release toxic smoke after combustion, foam thermal insulation boards have the advantages of being light weight; providing fire prevention, heat insulation, sound insulation and seismic resistance; being non-toxic and harmless; being able to be firmly bonded to the wall; and having a long service life [2]. But like ordinary concrete, the production and preparation of foam insulation boards also need to consume a lot of cement. The cement industry is one of the most important sources of greenhouse gas CO_2_ emissions, so in order to reduce the use of cement materials and carbon emissions, more and more experts and scholars are proposing to seek new cementitious materials to replace cement or reduce cement consumption to reduce carbon emissions. Construction waste has gradually entered people’s field of vision, and research has found that ultrafine grinding can help in replacing cement as an auxiliary cementitious material.

In recent years, scholars in China and abroad have explored and studied foam concrete and recycled micro powder. In 1998, Durack et al. [3] found through experimental research that the porosity of foam concrete directly determines the development of its compressive strength and thermal conductivity. In 2004, Singaporean scholar K. Ruiwen et al. [4] conducted research on the strength improvement in foam concrete, making the application of foam concrete more extensive. L. Y. Guan [5] carried out a detailed study on the macro mechanical properties of foam concrete, established a model of mechanical properties and pore structure, obtained a mix design theory with certain data support, and carried out engineering test verification on the prepared low-density foam concrete. The results showed that its working performance was excellent and met the requirements of relevant specifications for application. Y. Ji et al. [6] studied the influence of various mineral admixtures on the physical properties of foam concrete and found that the main role of mineral admixtures is to significantly reduce the number of harmful holes in foam concrete, thus improving the physical and mechanical properties of foam concrete. X. Y. Liu et al. [7] conducted research on the properties of recycled micro powder and the performance of recycled micro powder concrete through strength tests and plate tests. They found that the activity index of recycled micro powder was similar to that of mineral powder. After adding a small amount of recycled micro powder to concrete, the macroscopic performance of the concrete showed an improvement in early crack resistance and later compressive strength. S. H. Gao et al. [8] prepared recycled micro powder from C30 waste concrete and studied the influence of different recycled micro powder contents (0, 5%, 10%, 15%) on the mechanical properties of foam concrete. The results showed that the 28 d compressive strength of foam concrete increased first and then decreased with an increase in micro powder content. S. B. Yang [9] explored the influence of recycled micro powder content (0, 10%, 20%, 30%) on the water absorption, compressive strength and thermal conductivity of foam concrete. The results showed that recycled micro powder had little effect on the water absorption and thermal conductivity of foam concrete. However, with an increase in recycled micro powder content, the compressive strength of foam concrete first increased and then decreased, reaching the maximum value at 10% content. X. J. An et al. [10] set the replacement rate of recycled micro powder for cement at 0, 10%, 20%, 30% and 40% to prepare foam concrete and explored the influence of recycled micro powder on the performance of foam concrete. The results showed that with an increase in the replacement rate of recycled micro powder, the thermal conductivity of foam concrete first increased and then decreased. When the replacement rate of recycled micro powder was 8%, it reached the maximum value, and the thermal insulation performance was the worst. The 7 d compressive strength of foam concrete gradually decreased, and the 28 d compressive strength first increased slowly and then decreased rapidly. S. Y. Chen et al. [11] carried out research on the preparation of recycled micro powder foam concrete by the physical foaming process. Adding fiber to foam concrete could increase the proportion of small holes in foam concrete, and its compressive strength and flexural strength were improved.

In order to reduce the use of cement materials and reduce carbon emissions, recycled micro powder can be added to prepare foam concrete. However, at present, many studies only explore the impact on the performance of foam concrete by mixing recycled brick powder or recycled concrete powder separately. In the process of recycling construction waste (mostly waste concrete and waste clay bricks), due to the complex conditions of the construction site, it is difficult to completely achieve classified collection, which leads to the existence of recycled materials in mixed proportions, and the application of recycled micro powder in actual projects often uses mixed proportions. The different mixing proportions of recycled micro powder lead to a difference in the performance of foam concrete. Therefore, it is particularly important to study the mixing proportion of recycled micro powder. At the same time, the performance of foam concrete is mainly determined by its internal pore structure, so excellent foam is very important for foam concrete [12]. The performance of foam depends on the foaming agent itself. A foaming agent with excellent performance can produce foam with uniform size distribution and high stability. Therefore, the foaming agent is the key technology in the preparation of foam concrete [13,14,15]. In recent years, bio-based blowing agents have garnered widespread attention due to their eco-friendly properties. Among these, bio-based blowing agents produced by microorganisms (such as yeast) not only offer advantages such as strong foaming capacity, small pore size, and high foam stability but also feature a simple production process and are both environmentally friendly and cost-effective [16,17,18].

To sum up, existing research mainly focuses on the application of single recycled micro powder in foam concrete, but the synergistic effect of hybrid recycled micro powder still lacks a systematic discussion. To determine a reasonable replacement ratio of recycled micro powder, preliminary experiments were conducted prior to a formal study. The effects of different replacement ratios of recycled brick powder (30%, 50%, and 70%) on the compressive strength of A03 foamed concrete were compared. The corresponding compressive strengths were 0.54 MPa and 0.38 MPa for 30% and 50%, respectively, while the specimens could not be formed at a 70% replacement ratio. These results indicate that compressive strength decreases significantly with an increasing replacement ratio. In comparison, the 30% replacement level maintains relatively good mechanical performance while achieving a high utilization of solid waste. Therefore, 30% was selected as the fixed replacement ratio for the subsequent systematic investigation. On this basis, in this paper, 30% recycled brick powder, recycled concrete powder and mixed recycled micro powder (recycled brick powder:recycled concrete powder ratio is 4:6 and 6:4) were added to replace cement to prepare A03 (note: A03 is 300 ± 50 kg/m^3^) density-grade foam concrete according to the mass fraction, and its influence on the dry density and compressive strength of foam concrete was explored. The effects of different mixing ratios (recycled brick powder:recycled concrete powder: 2:8, 4:6, 6:4, 8:2) on the macro properties (compressive strength, drying shrinkage and thermal conductivity) and microstructure (hydration products and micro morphology) of the foam insulation board were systematically studied, aiming to clarify the synergistic mechanism of hybrid recycled micro powder in the foam insulation board from the aspects of performance and mechanism and finally determine its optimal proportion in the field of insulation.

## 2. Materials and Methods

### 2.1. Materials

**Figure 1:**
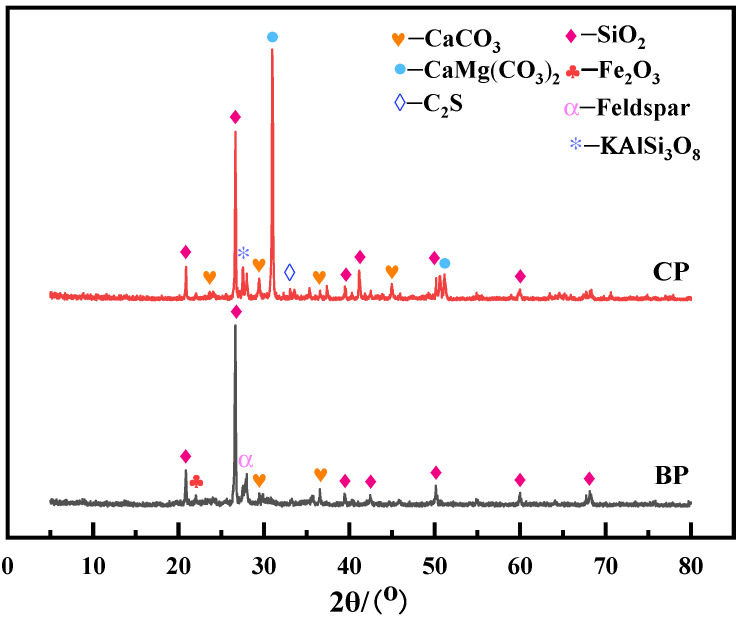
XRD diagram of recycled micronized powder.

Regenerated micro powder: The raw materials of construction waste are provided by Tianjin Zhongtang Industrial Park and screened in the laboratory. The recycled aggregate selected is ground by a cement test mill, and then the recycled fine powder with a particle size less than 0.075 mm is obtained by screening. The recycled brick powder needs to be ground for 20 min, and the recycled concrete powder needs to be ground for 40 min. The main chemical components and XRD patterns are shown in Table 1 and Figure 1, respectively. According to Table 1 and Figure 1, the chemical composition of regenerated micro powder is mainly oxides of silicon, aluminum, calcium, iron, magnesium and other elements. The mineral composition of both consists of quartz (SiO_2_), with a small amount of feldspar and hematite (Fe_2_O_3_) in the recycled brick powder. The recycled concrete powder mainly consists of dolomite (CaMg(CO_3_)_2_) and a small amount of limestone (CaCO_3_).

Cement: P∙O 42.5-grade ordinary Portland cement from Jinyu Cement Co., Ltd. (Tianjin, China). The initial setting time is 140 min, the final setting time is 250 min, the compressive strength at 28 days is 52.8 MPa, and the flexural strength is 8.8 MPa.

Foaming agent: In the laboratory, yeast was used as the raw material and combined with anionic and cationic surfactants to prepare a composite microbial foaming agent. Both surfactants were supplied by Tianjin Guangfu Fine Chemical Research Institute (Tianjin, China). The prepared foaming agent exhibited a foaming ratio of 28, a foam density of 48 kg/m^3^, a settlement distance of 2 mm within 1 h, and a bleeding rate of 27% after 1 h. Compared with the reported bleeding rates of 45–83% for foaming agents in the literature, the foaming agent prepared in this study shows relatively good performance [19].

Other concrete admixture: (1) Water reducing agent—Polycarboxylate water reducing agent. (2) Early strength agent—Anhydrous sodium sulfate, analytical grade, produced by Tianjin Guangfu Fine Chemical Research Institute (Tianjin, China). (3) Toughening agent—Basalt fiber 6 mm long, produced by Changsha Ningxiang Building Materials Factory (Changsha, China).

### 2.2. Test Scheme

(1) Recycled micro powder foam concrete: In a previous experimental study, it was found that the compressive strength of foam concrete prepared with 30% recycled micro powder had a low decline. Therefore, 30% recycled brick powder, recycled concrete powder and hybrid recycled micro powder (the mass ratio of BP and CP was 4:6 and 6:4) were added respectively to replace cement to prepare A03 density-grade foam concrete. After preparation was completed and the concrete reached the curing age, the influence of hybrid recycled micro powder on the performance of A03 density-grade foam concrete was explored by testing its macro performance (dry density, compressive strength).

**Figure 2:**
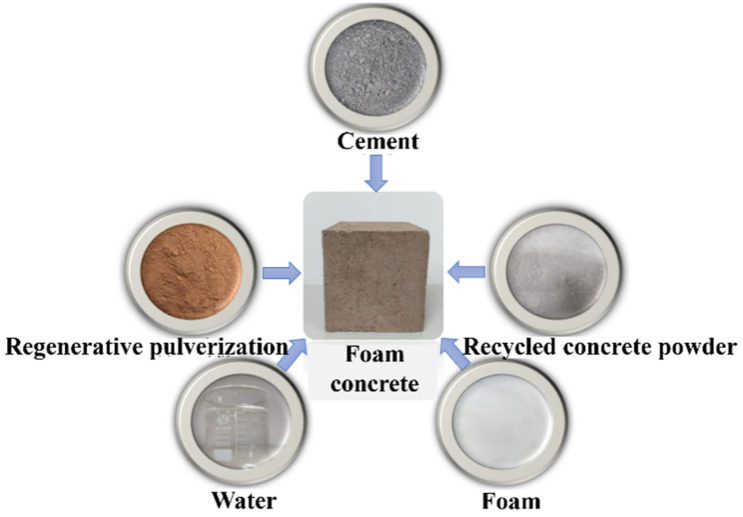
Macro morphology of recycled micro powder foam concrete molding materials and specimens after molding.

First, a self-made microbial foaming agent was prepared in the laboratory for standby; then the cement and recycled micro powder were added into a mixer according to the design mix proportion (see Table 2) to mix them evenly; then water and a water reducer were added to fully mix them; and then a cement foaming machine was used to prepare foam. After the preparation of foam, it was immediately added into the mixed slurry of cement and recycled micro powder to mix them for 3 min; after mixing them evenly, the mixture was quickly poured into a 100 mm × 100 mm × 100 mm mold; after 6 h, film covering for curing was performed; after 24 h, it was demolded; and then it was wrapped with plastic wrap at room temperature for curing for 28 days. After curing, it was dried (65 °C) to constant weight. The macro morphology of recycled micro powder foam concrete molding materials and specimens after molding is shown in Figure 2.

**Figure 3:**
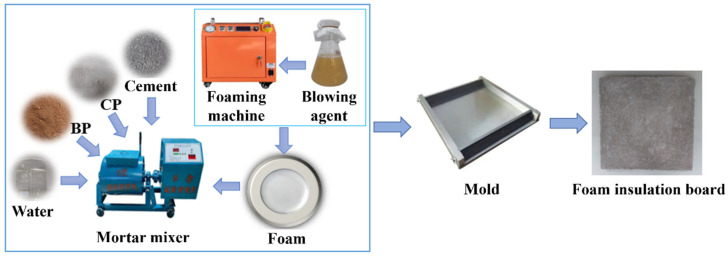
Mixed recycled micronized foam insulation board preparation flow chart.

(2) Hybrid recycled micro powder foam insulation board: In order to obtain the optimal ratio of recycled brick powder and recycled concrete powder in hybrid recycled micro powder applied in the field of building insulation, a test was conducted based on the preparation of hybrid recycled micro powder with a dosage of 30% (in which the mass ratios of BP and CP were 2:8, 4:6, 6:4, and 8:2) based on a microbial foaming agent and foam insulation board (density was 250 ± 10 kg/m^3^). The foam insulation board was prepared according to the set mix proportion (see Table 3). The specific preparation process is shown in Figure 3.

The effect of m (BP):m (CP) on the macro performance (compressive strength, drying shrinkage and thermal conductivity) and microstructure (hydration products and micro morphology) of the foam insulation board was studied by adding 30% of hybrid recycled micro powder and using different m (BP):m (CP) (2:8, 4:6, 6:4, 8:2) ratios.

### 2.3. Test Method

#### 2.3.1. Testing Methods of Recycled Micro Powder Foamed Concrete

The dry density and compressive strength of the cured recycled micro powder foam concrete are in accordance with “Foam Concrete” (JC/T 266-2011). Specifically, for foam concrete of density class A03, dry density is ≤300 kg/m^3^, and compressive strength ranges from 0.3 to 0.7 MPa.

#### 2.3.2. Testing Methods for Mixed Recycled Micronized Foam Insulation Board

(1) The compressive strength of the hybrid recycled micro powder foam insulation board was tested according to “Test Methods for Inorganic Thermal Insulation Products” (GB/T 5486-2008). Compressive strength tests were conducted using 100 mm × 100 mm × 100 mm cubic specimens, and the average value of 6 test pieces was taken as the test result.

(2) The drying shrinkage of the hybrid recycled micro powder foam insulation board (soaking time is 72 h) was tested according to “the Performance Method of Autoclaved Aerated Concrete” (GB/T 11969-2020). Test specimens measuring 40 mm × 40 mm × 160 mm were subjected to a drying shrinkage test under environmental conditions of (20 ± 2) °C and (60 ± 5)% relative humidity, and the average value of 3 test pieces was taken as the test result.

(3) The thermal conductivity of the hybrid regenerated micro powder foam insulation board was tested according to “Determination of Steady State Thermal Resistance and Related Properties of Thermal Insulation Materials—Protective Hot Plate Method” (GB/T 10294-2008). The test specimens measure 300 mm × 300 mm × 30 mm and were tested under steady-state thermal equilibrium conditions. The test results are based on the arithmetic mean of three specimens.

(4) The central part of the broken foam concrete specimen was taken as the sample, at least three samples from each group of test specimens were selected, and the hydration products were analyzed by an Ultima IV X-ray diffractometer (Rigaku Corporation, Tokyo, Japan); JEC-3000FC AUTO FINE COATER full-automatic ion sputtering instrument (JEOL, Tokyo, Japan) was used to spray gold and coating on the samples. Gold was sprayed at 10 mA. After spraying gold, the microstructure was analyzed by a JSM-7800F scanning electron microscope (Guangzhou Yide Precision Scientific Instruments Co., Ltd., Guangzhou, China). During the test, the accelerating voltage was set to 5 kV, the working distance was approximately 8–10 mm, and observations were made using the secondary electron (SE) imaging mode. For each group of samples, at least three test specimens were selected for testing, and representative areas were examined.

## 3. Results and Discussion

### 3.1. Recycled Micro Powder Foamed Concrete

#### 3.1.1. Dry Density

**Figure 4:**
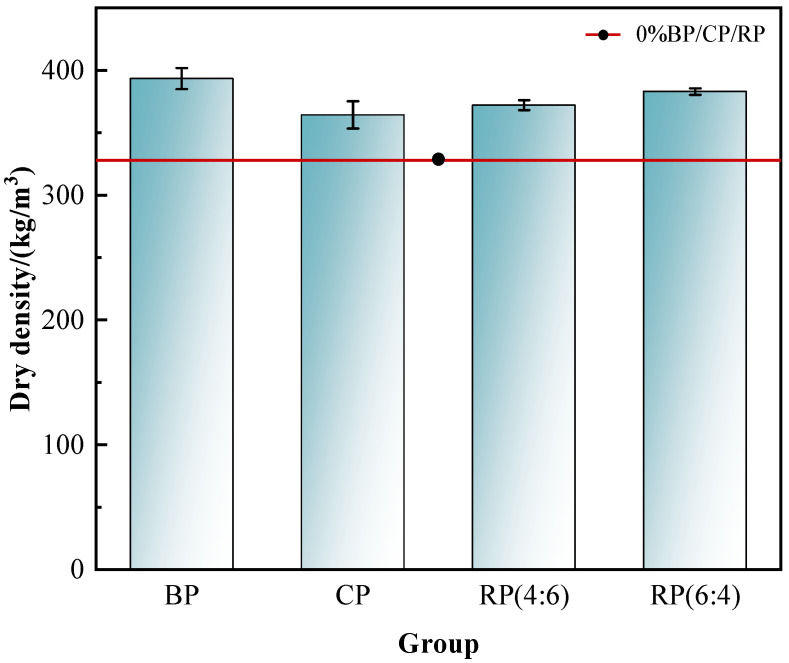
Influence of recycled micro powder category on dry density of A03-grade foam concrete.

The change rule of the dry density of A03-grade foam concrete mixed with 30% BP, CP, and RP (4:6, 6:4) is shown in Figure 4.

It can be seen from Figure 4 that the influence of four recycled micro powders on the dry density of A03 foam concrete is as follows: recycled brick powder > hybrid recycled micro powder (6:4) > hybrid recycled micro powder (4:6) > recycled concrete powder. For A03-grade foam concrete, when the mixing amount of recycled brick powder, recycled concrete powder and hybrid recycled micro powder (4:6 and 6:4) is 30%, dry density is 393 kg/m^3^, 364 kg/m^3^, 372 kg/m^3^ and 383 kg/m^3^ respectively, which are 19.8%, 11.0%, 13.4% and 16.8% higher than that of the control group, at 328 kg/m^3^.

Because the specific surface area of recycled brick powder is about 856 m^2^/kg, recycled concrete powder is about 745 m^2^/kg, and cement is about 375 m^2^/kg, the specific surface area of cement is much smaller than that of recycled brick powder and recycled concrete powder. At the same time, the connected pores of cement are mainly distributed below 10 nm, but the pore size of recycled brick powder is also concentrated in the range of 10~60 nm. The larger the specific surface area and pore size of the material, the stronger its water absorption [20]. For A03 density-grade foam concrete prepared with 30% recycled micro powder, because compared to recycled concrete powder and mixed recycled micro powder (4:6, 6:4), recycled brick powder has a larger specific surface area and stronger water absorption, the impact on the dry density of foam concrete is more significant. Therefore, the dry density of foam concrete prepared by mixing recycled brick powder alone is the highest, while the impact of recycled concrete powder is the smallest. For mixed recycled micro powders (4:6, 6:4), when the mixing ratio is 6:4, the content of recycled brick powder is higher, so its water absorption is also stronger. Therefore, the dry density of A03 foam concrete prepared with recycled micro powder mixed at 6:4 is higher than that of foam concrete prepared at 4:6.

#### 3.1.2. Effect of Recycled Micro Powder on the Compressive Strength of A03-Grade Foam Concrete

**Figure 5:**
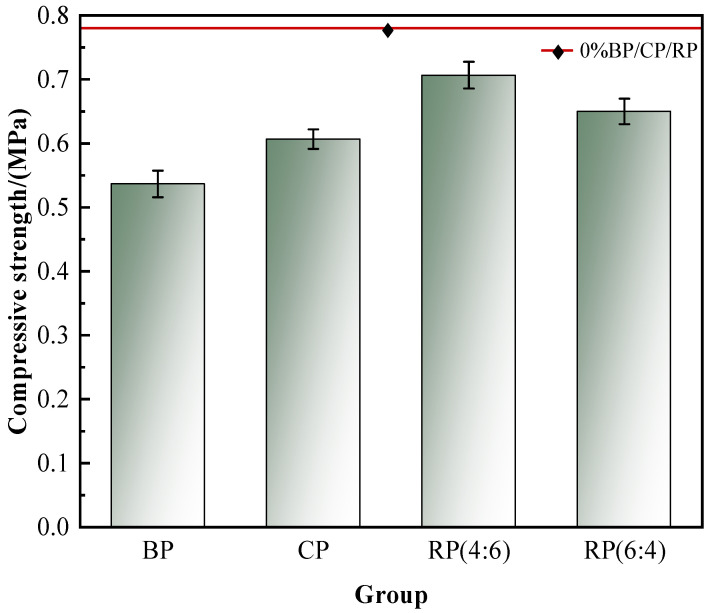
Effect of recycled micro powder on compressive strength of A03-grade foam concrete.

The change rule of the compressive strength of A03-grade foam concrete mixed with 30% BP, CP, and RP (4:6, 6:4) is shown in Figure 5.

According to Figure 5, the degree of influence of the four types of recycled micro powders on their compressive strength is in the following order: recycled brick powder > recycled concrete powder > mixed recycled micro powders (6:4) > mixed recycled micro powders (4:6). When 30% recycled brick powder, recycled concrete powder and mixed recycled micro powder (4:6 and 6:4) were added, the compressive strength of A03-grade foam concrete was lower than the 0.78 MPa of the control group, and the specific values were 0.54 MPa, 0.61 MPa, 0.70 MPa and 0.65 MPa, respectively, and decreased by 31%, 22%, 10% and 17% compared with the control group. Among them, mixed recycled micro powder (4:6) has the smallest impact on the compressive strength of A03 foam concrete, so A03 foam concrete mixed with mixed recycled micro powder (4:6) has the highest compressive strength, while the compressive strength of recycled brick powder alone is the lowest. X. J. An et al. [10] showed that the compressive strength of foam concrete mixed with 30% recycled micro powder was about 14% lower than that of the control group. The compressive strength of foamed concrete with 30% mixed recycled micro powder (4:6) prepared in this study decreased by 10%, indicating that the combined use of a specific proportion of mixed recycled micro powder can produce a synergistic reinforcement effect, which is better than that of a single recycled micro powder.

In the mixed recycled micro powder (4:6, 6:4), there are three types of cementitious materials in the entire cementitious system, cement, recycled brick powder, and recycled concrete powder, and the particle size of these three cementitious materials varies. Therefore, when recycled brick powder and recycled concrete powder are mixed, they can effectively fill the pores between the matrix and enhance the compactness of the matrix. At the same time, the hybrid regenerated micro powder contains a large amount of CaO and a small amount of CaCO_3_ and C_2_S, which can supplement the calcium source for the cementitious system and carry out the secondary hydration reaction [21,22]. In addition, the high pozzolanic activity of the mixed regenerated micro powder will also stimulate the consumption of Ca(OH)_2_ newly generated in the cementitious system, which will promote the hydration reaction between cement clinker and cement to a certain extent [23]. Therefore, the hybrid recycled micro powder can not only play a good role in physical filling but also participate in the hydration process. Therefore, the compressive strength of foam concrete prepared by hybrid recycled micro powder is higher than that of foam concrete mixed with recycled brick powder and recycled concrete powder alone.

### 3.2. Mixed Recycled Micronized Foam Insulation Board

#### 3.2.1. Macroscopic Morphology

**Figure 6:**
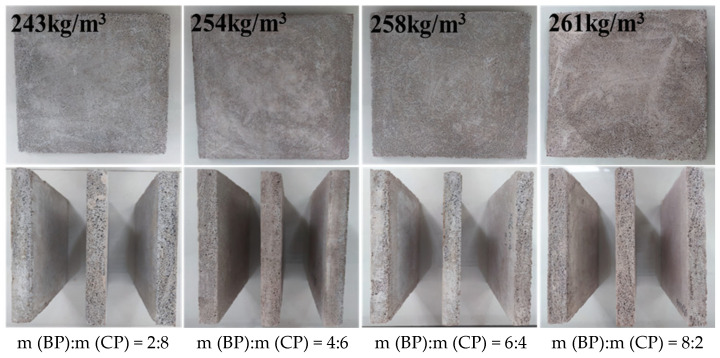
Mixed recycled micronized foam insulation board.

The mixed regenerated micro powder foam insulation board with different 30% m (BP):m (CP) proportions prepared by a microbial foaming agent is shown in Figure 6.

#### 3.2.2. Compressive Strength of Mixed Recycled Micronized Foam Insulation Board

**Figure 7:**
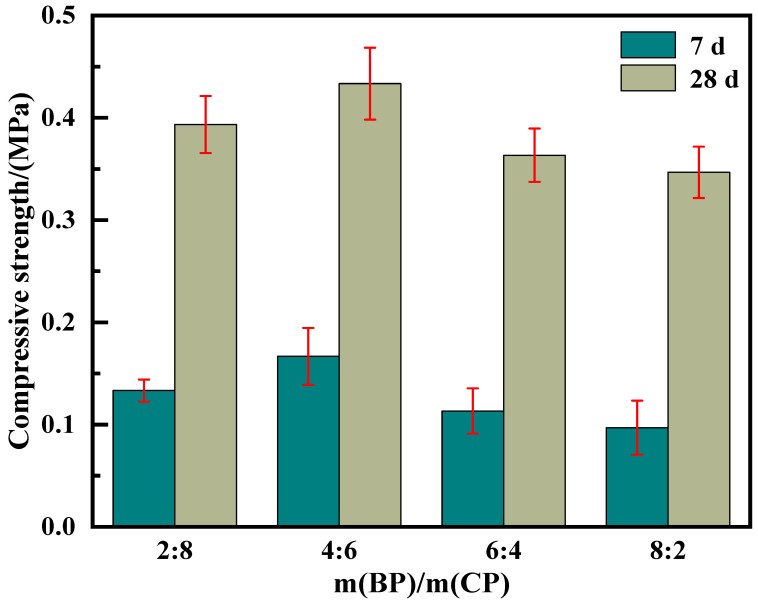
Compressive strength of mixed recycled micronized foam insulation board.

The influence of recycled micro powder content mixed using recycled brick powder and recycled concrete powder with different mass proportions on the compressive strength of the foam insulation board is shown in Figure 7.

It can be seen from Figure 7 that at an age of 7 days, the strength of the mixed regenerated micro powder foam insulation board with four proportions shows little difference. As the maintenance age increases, the strength difference gradually increases after reaching 28 days. When the ratio of m (BP):m (CP) is 4:6, the strength reaches its maximum value (0.43 MPa), and when the ratio is 8:2, the strength drops to its lowest value (0.35 MPa). This is mainly because mixed recycled micro powders, as auxiliary cementitious materials, usually have a diluting effect, physical filling effect, and volcanic ash effect on cement hydration [24]. After mixed with recycled micro powder, the amount of cement is reduced, which limits the development of the early strength of the foam insulation board. Moreover, the early volcanic ash activity of mixed regenerated micro powders is relatively low, and physical filling plays a dominant role, resulting in the slow development of the hydration degree. Therefore, at an age of 7 days, its strength mainly relies on the hydration reaction of cement, and the hydration effect of mixed recycled micro powder is relatively small, so its 7-day strength is not significantly different.

As hydration continues, the volcanic ash activity of the mixed regenerated micro powder will consume the newly generated Ca(OH)_2_ in the cementitious system, which will to some extent promote the hydration reaction of the composite material. C_2_S and other hydration products in recycled concrete powder will also participate in the hydration system, undergo secondary hydration reactions, provide Ca^2+^ for the cementitious system, and act as crystal embryos [25]. The aluminum salt phase contained in the recycled brick powder will also stimulate the hydration nucleation effect [26]. Therefore, after reaching an age of 28 days, the compressive strength difference in the foam insulation board made of different m (BP):m (CP) hybrid recycled micro powders will begin to appear. Meanwhile, the early volcanic ash activity of recycled brick powder is lower than that of recycled concrete powder, and the fineness of recycled concrete powder is higher, so its 28-day volcanic ash activity is higher than that of recycled brick powder. Therefore, when m (BP):m (CP) is 6:4 and 8:2, the strength of the foam insulation board is low. When m (BP):m (CP) is 4:6, due to the good gradation ratio of recycled micro powder and recycled concrete powder, their physical filling and hydration effects combine to fill the small pores in the cementitious system and compensate for some hydration reactions. Therefore, when m (BP):m (CP) is 4:6, the strength of the hybrid regenerated micro powder foam insulation board is the highest.

#### 3.2.3. Drying Shrinkage

**Figure 8:**
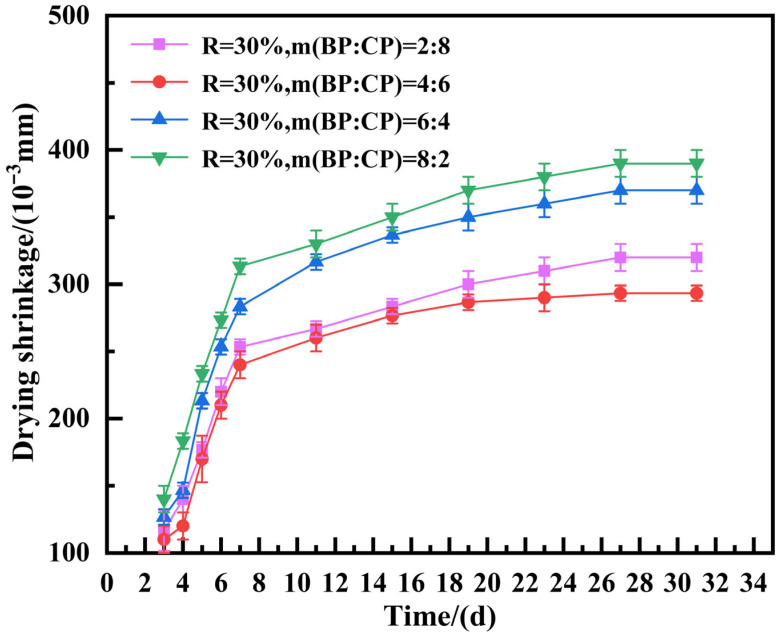
Drying shrinkage of mixed recycled micronized foam insulation board.

The drying shrinkage of the foam insulation board prepared with 30% recycled micro powder in different proportions was tested. The drying shrinkage of the board was measured for 3 days, 4 days, 5 days, 6 days, 7 days, 11 days, 15 days, 19 days, 23 days, 27 days, 31 days and 35 days (constant weight). The results are shown in Figure 8.

It can be seen from Figure 8 that the drying shrinkage of the foam insulation board prepared with different proportions of hybrid regenerative micro powder decreases slowly at first and then increases rapidly with an increase in m (BP):m (CP) in hybrid regenerative micro powder. Shrinkage growth is relatively fast in the first 7 days and slowly increases after 11 days. By around 27 days, as the mass tends to be constant, drying shrinkage also stabilizes and basically stops growing. Meanwhile, by comparing the drying shrinkage values of mixed regenerated micro powder insulation boards under different m (BP):m (CP) ratios, it was found that drying shrinkage was smaller when m (BP):m (CP) ratios were 2:8 and 4:6. When m (BP):m (CP) is 4:6, the minimum is only 0.29 mm. When this ratio increases to 6:4, its drying shrinkage begins to significantly increase, reaching a maximum value of 0.39 mm at an m (BP):m (CP) ratio of 8:2. Through analysis, it can be concluded that when the ratio of m (BP) to m (CP) is 6:4 and 8:2, the mixed recycled micro powder is unevenly distributed in the cement matrix, causing the uneven hydration of the composite cementitious system and increasing drying shrinkage [27].

In addition, because of the porous characteristics of foam concrete, high porosity will provide a convenient channel for the loss of water inside the material. In the early stage of hydration, a large amount of water on the external surface evaporates due to its porous nature, resulting in the incomplete hydration of the matrix. The low early activity of the regenerated brick powder prevents timely solidification and causes defoaming, which further forms larger open pores. These pores cause a large amount of water loss, forming a vicious cycle and further increasing its drying shrinkage. Therefore, in general, the surface pore diameter of the foam insulation board is larger than its internal pore diameter, and early shrinkage is larger. When a lot of moisture is lost, the matrix slowly becomes stable, and its drying shrinkage will also tend to be flat. So when the content of recycled brick powder is high, the hydration rate slows down, and the surface water loss is faster, causing defoaming and the formation of many large pore connected pores, resulting in larger drying shrinkage when m (BP):m (CP) is 6:4 and 8:2.

#### 3.2.4. Thermal Conductivity

**Figure 9:**
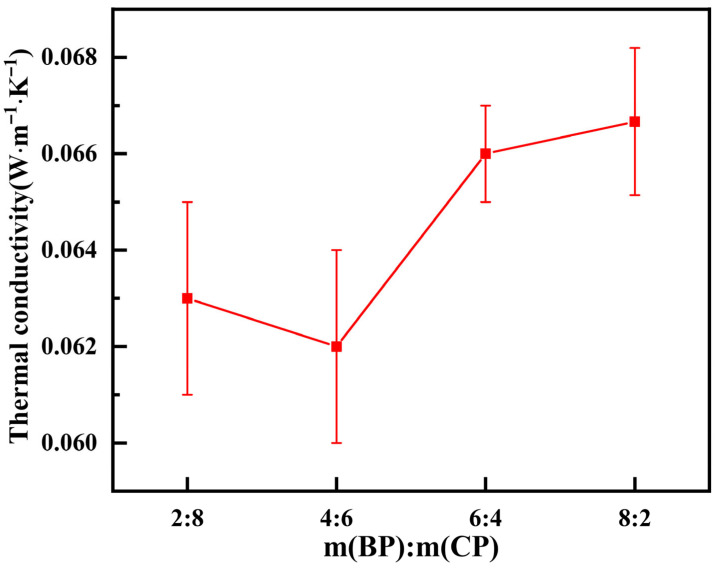
Thermal conductivity of mixed recycled micronized foam insulation board.

The test results of the thermal conductivity of the hybrid recycled micro powder foam insulation board mixed with recycled brick powder and recycled concrete powder in different mass proportions are shown in Figure 9.

It can be seen from Figure 9 that the thermal conductivity of the foam insulation board prepared by mixing different proportions of recycled micro powder is low, and thermal insulation performance is good. Thermal conductivity first decreases and then increases with an increase in m (BP):m (CP). When m (BP):m (CP) is 4:6, the lowest thermal conductivity is 0.062 W∙m^−1^∙K^−1^. This is because at this time, the gradation of regenerated micro powder is better, and the physical filling effect is maximized. At the same time, the content of recycled concrete powder is relatively high, and the CaO content in recycled concrete powder is higher than that in recycled brick powder. It also contains a small amount of CaCO_3_ and C_2_S, which can supplement the calcium source for the entire cementitious system and carry out secondary hydration reactions [21,22]. The hydration of the matrix is relatively complete, and the defoaming phenomenon during the setting process is low. Therefore, the porosity is higher after molding, and there are more closed pores. As is known, the pore distribution of foam concrete directly affects thermal insulation and heat storage performance, while the thermal conductivity of the gas phase in porous materials is significantly lower than that of the solid phase, and the thermal conductivity of closed pores is significantly lower than that of through pores. Moreover, the more gas–solid interfaces, the slower the radiation conduction between the interfaces and the lower the heat conduction efficiency of materials [28]. Therefore, the higher the porosity, the more closed pores there are and the lower the thermal conductivity of the material. So as m (BP):m (CP) increases, thermal conductivity gradually increases. This is because the internal structure of recycled brick powder is relatively loose, and the early low activity and incomplete hydration lead to the formation of many large open pores in the formed specimens, which in turn cause a slight increase in thermal conductivity.

#### 3.2.5. Hydration Product

**Figure 10:**
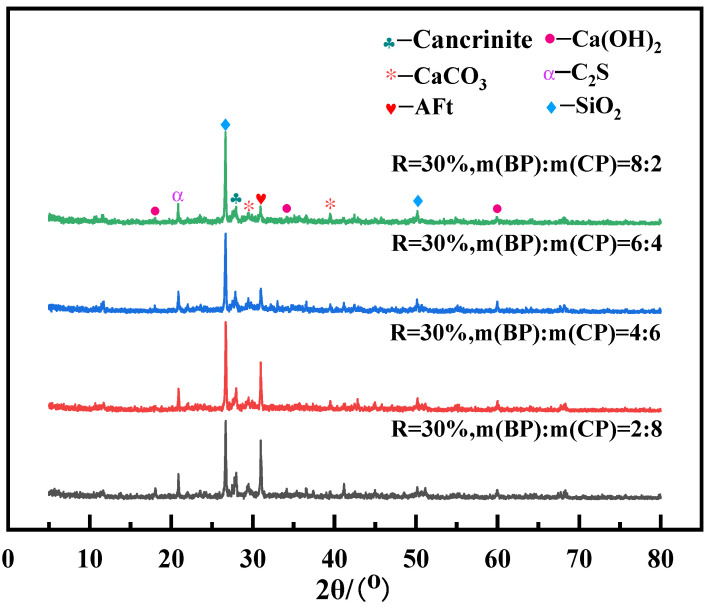
XRD pattern of mixed recycled micronized foam insulation board.

Figure 10 shows the 5°~80° XRD atlas of the foam insulation board prepared with 30% mixed regenerated micro powder with an m (BP):m (CP) content of 2:8, 4:6, 6:4, and 8:2 for 28 days of curing.

From Figure 10, it can be seen that at a 30% mixed recycled micro powder dosage, there is no significant difference in the hydration product phases of different m (BP):m (CP) ratios. The main phases are Ca(OH)_2_, C_2_S (dicalcium silicate), AFt (ettringite), Cancrinite (calcareous garnet), and CaCO_3_ (calcite). When m (BP):m (CP) is 2:8 and 4:6, the intensity of the characteristic diffraction peak of Cancrinite is high. Based on previous studies [29,30], it is speculated that the presence of Cancrinite and CaCO_3_ may play a role in promoting the formation and densification of the C-(A)-S-H gel. At the same time, under the two aforementioned ratios of recycled brick powder, the AFt diffraction peak is quite pronounced; its formation may, to some extent, serve to fill the pore structure, thereby having a positive effect on the material’s early-stage strength development. As m (BP):m (CP) increases, its SiO_2_ diffraction peak gradually strengthens. This is because the SiO_2_ content in the regenerated brick powder is relatively high, and the content of regenerated brick powder gradually increases, resulting in an increase in its SiO_2_ content.

#### 3.2.6. Microstructure

**Figure 11:**
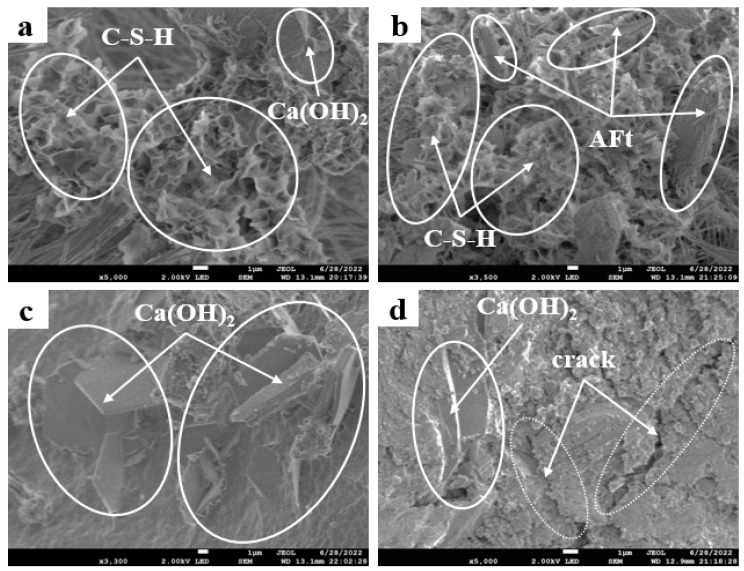
The 28 d SEM plots of mixed recycled micronized foam insulation boards: (**a**) m (BP):m (CP) = 2:8; (**b**) m (BP):m (CP) = 4:6; (**c**) m (BP):m (CP) = 6:4; (**d**) m (BP):m (CP) = 8:2.

The micro morphology of the hybrid regenerated micro powder foam insulation board with 30% m (BP):m (CP) ratios of 2:8, 4:6, 6:4, and 8:2 is shown in Figure 11.

It can be seen from Figure 11a–d that when m (BP):m (CP) is 2:8 and 4:6, more C-S-H gel is generated by hydration. At the same time, when m (BP):m (CP) is 2:8 and 4:6, more AFt is also generated in combination with XRD spectra; C-S-H gel is the main source of its strength, and AFt can also provide some early strength. Moreover, there is a difference in particle size between recycled concrete powder and cement. Compared with cement particles, recycled concrete powder has a good particle size distribution, with overall finer particles and lower angularity. Recycled concrete powder particles with better grading can be filled with cement particles to form a better grading, exerting a certain physical filling effect, filling some pores, and increasing the compactness of the entire cementitious system [31]. When m (BP):m (CP) is 6:4, the volcanic ash activity of early recycled concrete powder is higher than that of recycled brick powder. As the content of recycled brick powder increases, the content of recycled concrete powder decreases. The early volcanic ash activity of mixed regenerated micro powders will decrease, leading to a slower hydration reaction. Therefore, the content of C-S-H gel and AFt produced is reduced, and a large amount of Ca(OH)_2_ is enriched. The hydration distribution is uneven, resulting in a decrease in strength. When m (BP):m (CP) continues to increase to 8:2, the activity of hybrid regenerated micro powder further decreases, and the content of products mainly providing strength sources such as C-S-H gel is lower. It can be seen from Figure 11d that at this time, not only are the hydration products reduced, but also more microcracks appear, and the structure is relatively loose. The reason is that when the content of recycled brick powder is too high, due to its large specific surface area, it will first absorb the moisture in the cementitious system, resulting in the insufficient hydration of cement. In addition, the early activity of mixed recycled micro powder is low, the hydration process is slow, and the number of unhydrated particles increases. As a result, its structure is relatively loose, with microcracks and defects in interface connections. At the same time, due to an increase in unhydrated C_2_S, the bonding stress between the cement matrix and the incompletely hydrated particles deteriorates. The overall strength decreases.

## 4. Conclusions

(1) The influence degree of four recycled micro powders on the dry density of foam concrete is as follows: recycled brick powder > hybrid recycled micro powder (6:4) > hybrid recycled micro powder (4:6) > recycled concrete powder. The degree of influence on compressive strength is as follows: recycled brick powder > recycled concrete powder > mixed recycled micro powder (6:4) > mixed recycled micro powder (4:6). Under the conditions of this study, the compressive strength of foamed concrete prepared with mixed recycled micro powder is higher than that of foamed concrete prepared with recycled brick powder and recycled concrete powder alone, indicating that the two kinds of recycled micro powder may have synergistic effects on performance at a specific proportion.

(2) The compressive strength, drying shrinkage and thermal conductivity of the mixed recycled micro powder foam insulation board increased first and then decreased with an increase in the mass ratio of recycled brick powder to recycled concrete powder. When the ratio of mixed recycled micro powder is 4:6, there is a compressive strength of 0.43 MPa, a drying shrinkage of 0.29 mm, and a thermal conductivity of 0.062 W·m^−1^·K^−1^. All performance parameters meet the requirements of “Cement based foam Insulation Board” (JC/T 2200-2013).

(3) From the micro level, with an increase in the proportion of hybrid regenerated micro powder, hydration products such as C-S-H gel and AFt are gradually reduced, while the content of Ca(OH)_2_ and unhydrated particles are gradually increased, the matrix structure of the material is looser, and there are many microcracks at the interface. Therefore, its macroscopic performance declined.

(4) The specific surface area of recycled micro powder is larger than that of cement, and foam concrete has a porous structure. Therefore, the water absorption of low-density recycled micro powder foam concrete can be studied in the future. For foam concrete with high water absorption, we can try to reduce water absorption by adding a waterproof agent or generating hydrophobic substances to form a hydrophobic film.

## Figures and Tables

**Table 1:** Main chemical composition of cement and recycled bricks and concrete powder w/%.

Materials	SiO_2_	Al_2_O_3_	CaO	Fe_2_O_3_	MgO	SO_3_	Na_2_O	K_2_O
Cement	21.22	9.29	43.89	1.63	8.22	2.24	0.65	0.77
BP	36.79	10.99	19.38	5.36	3.99	0.91	1.16	2.27
CP	27.34	6.96	30.95	3.58	6.38	0.48	0.99	1.56

**Table 2:** A03-grade recycled micro powder foam concrete ratio.

Powder Content	Mix Proportion/(kg∙m^−3^)				
	Cement (kg)	BP/CP/RP (kg)	Foam (m^3^)	Water (kg)	Water Reducer/(kg)
m_BP/CP/RP_ 0%	300	0	1.67	135	0.00
m_BP_ 30%	210	90	1.67	135	0.45
m_CP_ 30%	210	90	1.67	135	0.30
m_RP(4:6)_ 30%	210	90	1.67	135	0.39
m_RP(6:4)_ 30%	210	90	1.67	135	0.42

**Table 3:** Mixing ratio of hybrid recycled micro powder foam insulation board.

m (BP):m (CP)		Mix Proportion/(kg∙m^−3^)						
	Cement (kg)	BP (kg)	CP (kg)	Foam (m^3^)	Water (kg)	Water Reducer/(kg)	Fiber (kg)	Na_2_SO_4_ (kg)
2:8	168	14.40	57.60	1.86	96	0.84	1.20	7.20
4:6	168	28.80	43.20	1.86	96	0.96	1.20	7.20
6:4	168	43.20	28.80	1.86	96	1.08	1.20	7.20
8:2	168	57.60	14.40	1.86	96	1.20	1.20	7.20

## Data Availability

The original contributions presented in this study are included in the article. Further inquiries can be directed to the corresponding authors.
